# Protein arginine methyltransferase 1 is a novel regulator of MYCN in neuroblastoma

**DOI:** 10.18632/oncotarget.11556

**Published:** 2016-08-23

**Authors:** Allison Eberhardt, Jeanne N. Hansen, Jan Koster, Louis T. Lotta, Simeng Wang, Emmett Livingstone, Kun Qian, Linda J. Valentijn, Yujun George Zheng, Nina F. Schor, Xingguo Li

**Affiliations:** ^1^ Department of Pediatrics, University of Rochester School of Medicine and Dentistry, Rochester, New York, 14642, USA; ^2^ Department of Oncogenomics, Academic Medical Center, University of Amsterdam, 1105 AZ Amsterdam, The Netherlands; ^3^ Department of Pharmaceutical and Biomedical Sciences, College of Pharmacy, The University of Georgia, Athens, Georgia 30602, USA

**Keywords:** neuroblastoma, MYCN, PRMT1, arginine methylation, protein stability

## Abstract

Amplification or overexpression of MYCN is associated with poor prognosis of human neuroblastoma. We have recently defined a MYCN-dependent transcriptional signature, including protein arginine methyltransferase 1 (PRMT1), which identifies a subgroup of patients with high-risk disease. Here we provide several lines of evidence demonstrating PRMT1 as a novel regulator of MYCN and implicating PRMT1 as a potential therapeutic target in neuroblastoma pathogenesis. First, we observed a strong correlation between MYCN and PRMT1 protein levels in primary neuroblastoma tumors. Second, MYCN physically associates with PRMT1 by direct protein-protein interaction. Third, depletion of PRMT1 through siRNA knockdown reduced neuroblastoma cell viability and MYCN expression. Fourth, we showed that PRMT1 regulates MYCN stability and identified MYCN as a novel substrate of PRMT1. Finally, we demonstrated that mutation of putatively methylated arginine R65 to alanine decreased MYCN stability by altering phosphorylation at residues serine 62 and threonine 58. These results provide mechanistic insights into the modulation of MYCN oncoprotein by PRMT1, and suggest that targeting PRMT1 may have a therapeutic impact on MYCN-driven oncogenesis.

## INTRODUCTION

Amplification of *MYCN* oncogene has proven to be a major negative prognostic biomarker for neuroblastoma, an aggressive childhood cancer with overall survival of less than 50% for high-risk disease [1]. Therefore, *MYCN* gene amplification and subsequent overexpression represent attractive therapeutic targets for treatment of neuroblastoma. However, similar to the case for other transcription factors, strategies for modulation of MYCN function itself have proved challenging. Recently, a plethora of data has been generated regarding the oncogenic functions of MYCN in neuroblastoma [2]. Translation of this wealth of information into novel, effective, molecularly-targeted therapies is urgently needed.

We have previously defined a MYCN-dependent transcriptional signature that identifies a subset of patients with high-risk disease whose tumors have elevated MYCN protein levels [3]. The gene protein arginine methyltransferase 1 (*PRMT1*) was identified as a MYCN direct target gene and high levels of *PRMT1* mRNA correlated with poor prognosis in a series of 88 primary neuroblastoma tumors. PRMT1 is the predominant PRMT in mammalian cells and its activity accounts for 90% of all arginine methylation reactions in human cells [4]. Protein arginine methylation has been implicated in signal transduction, gene transcription, DNA repair and mRNA splicing, among other functions and aberrant expression of PRMT1 has been found in various types of cancer [4]. However, the underlying mechanisms by which PRMT1 contributes to oncogenesis are still largely unknown. We and others have recently shown that methylation of both histone and non-histone proteins by PRMT1 is essential for its function in many tissues [5–6]. Taken together, these data suggest that targeting PRMT1-mediated post-translational modification of proteins may provide a compelling therapeutic strategy for cancers, including neuroblastoma.

In this study, we identify PRMT1 as a novel essential regulator of MYCN and its level as a significant independent prognostic marker in primary neuroblastoma tumors. Mechanistically, we demonstrate a physical and functional interaction between PRMT1 and MYCN. PRMT1 methylates MYCN and controls the stability of MYCN through cross-regulation between methylation at arginine 65 (R65) and phosphorylation at conserved residues serine 62 (pS62) and threonine 58 (pT58). Our results highlight the potential of PRMT1 as a drug target for neuroblastoma and other cancers driven by the MYCN oncoprotein.

## RESULTS AND DISCUSSION

### *PRMT1* mRNA expression as a significant independent prognostic marker in primary neuroblastomas

We have previously identified PRMT1 within the functional MYCN-157 signature that characterizes neuroblastoma tumors with *MYCN* amplification, as well as tumors with high MYCN protein levels without *MYCN* amplification. In line with its relationship to MYCN, we demonstrated that high expression of *PRMT1* is associated with a poor prognosis in a series of 88 primary neuroblastoma tumors [3]. We confirmed that high *PRMT1* mRNA levels are prognostic for worse outcome with the Kocak dataset (Supplementary Figure S1A), which includes a cohort of 476 neuroblastoma patients. Furthermore, *PRMT1* expression was statistically significantly higher in stage 4 tumors (*P* < 0.01, Supplementary Figure S1B). In addition, by using multivariate Cox regression models based on overall survival of a cohort of 122 neuroblastoma patients (an expanded cohort of the previously described 88-patient dataset), we found that *PRMT1* expression adds to the predictive power of established risk markers (*MYCN* status and tumor stage). We observed a hazard ratio for survival of 3.217 for *PRMT1* expression, similar to *MYCN* amplification with a hazard ratio for survival of 2.909 (Table 1). These results demonstrate that *PRMT1* transcript levels contribute to discrimination between neuroblastoma patients with favorable and unfavorable outcome, respectively, and indicate that *PRMT1* mRNA expression helps to predict patient prognosis independent of stage and *MYCN* amplification status. To prospectively test the link between PRMT1 and neuroblastoma patient outcome, orchestrated participation of multiple institutions is required to achieve statistically useful numbers of patient samples of this tumor with an incidence of only 1:100,000 children [1]. We have therefore partnered with the Children's Oncology Group (COG) and obtained neuroblastoma tissue microarrays (TMAs) (see below). Future prospective studies will be needed to further evaluate the prognostic significance of PRMT1 expression in neuroblastoma patients.

**Table 1:** Multivariate Cox regression analysis based on overall survival considering *MYCN* status (amplified versus normal), stage (1-2, 4S versus 3-4; 1-3, 4S versus 4) and *PRMT1* expression

Variable	Hazard ratio (95% CI)	*P* value
**Univariate**		
*PRMT1* levels (high or low)	5.218 (2.709–10.05)	7.89E-07
*MYCN* amplification (yes or no)	5.602 (2.902–10.82)	2.83E-07
Stage (1, 2, 4S vs. 3, 4)	34.54 (4.727–252.4)	4.81E-04
Stage (1, 2, 3, 4S vs. 4)	19.55 (5.968–64.03)	9.05E-07
**Multivariate**		
*PRMT1* levels (high or low)	3.217 (1.479–6.999)	0.00321*
*MYCN* amplification (yes or no)	2.909 (1.336–6.334)	0.00716*
*PRMT1* levels (high or low)	3.623 (1.874–7.005)	1.30E-04*
Stage (1, 2, 4S vs. 3, 4)	27.141 (3.690–199.610)	0.00118*
*PRMT1* levels (high or low)	2.662 (1.367–5.181)	0.00397*
Stage (1, 2, 3, 4S vs. 4)	14.531 (4.334–48.721)	1.45E-05*

* Independent.

### PRMT1 protein expression correlates with MYCN levels in primary neuroblastoma tumors

**Figure 1:**
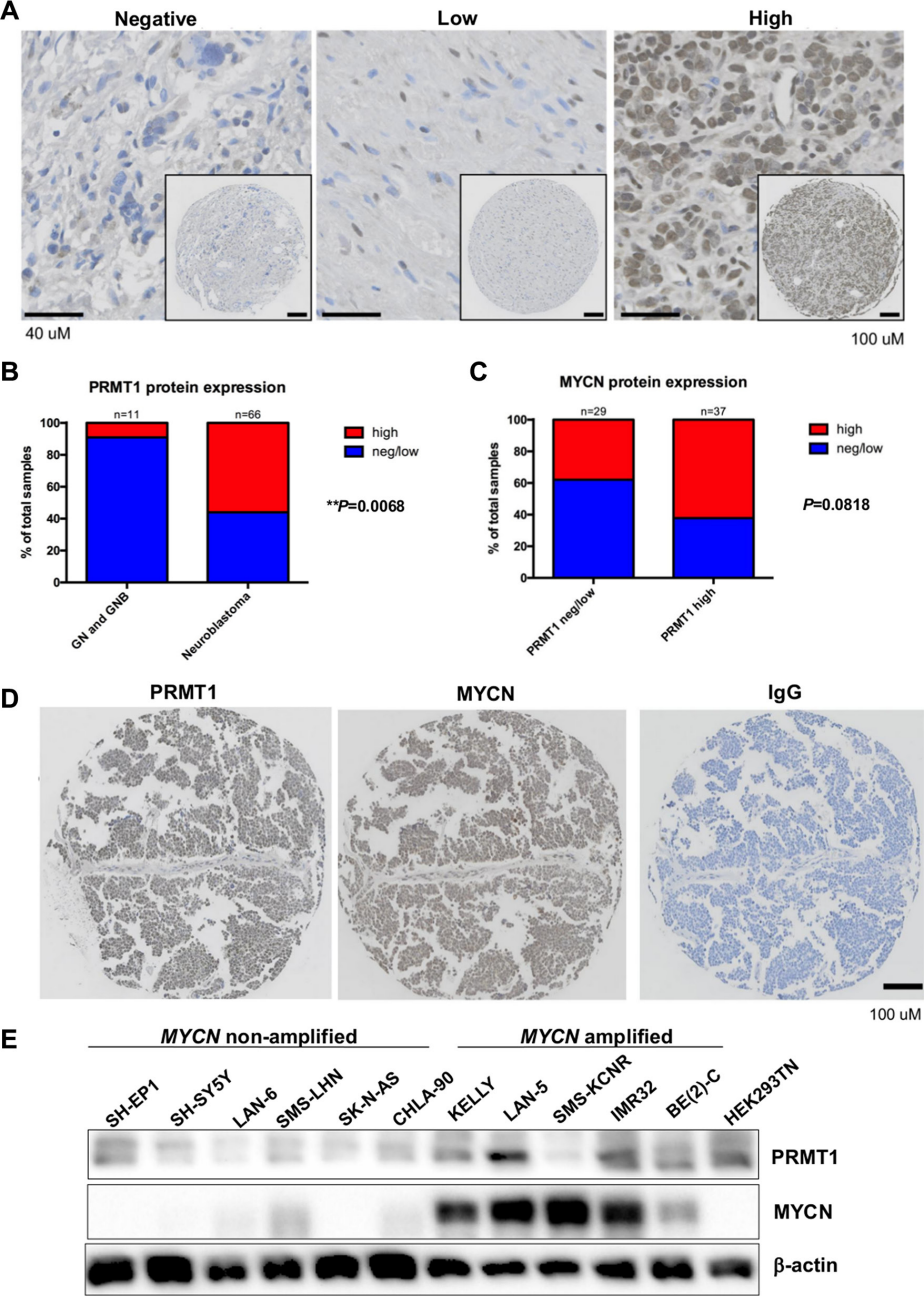
MYCN and PRMT1 correlations in neuroblastomas (**A**) Immunostaining for PRMT1 in neuroblastoma. (**B**) PRMT1 protein expression in ganglioneuroma (GN)/ganglioneuroblastoma (GNB) and neuroblastoma (NB) patients. **P < 0.01. (**C**) Bar graph shows case distribution of MYCN levels by PRMT1 levels. (**D**) Immunostaining for PRMT1 and MYCN in neuroblastoma. (**E**) Western blot of whole cell lysate from neuroblastoma cell lines with the indicated antibodies.

Our previous observation that MYCN activates transcription of *PRMT1* [3] prompted us to examine whether there is a correlation between MYCN levels and PRMT1 expression. We first investigated the relationship between MYCN and PRMT1 mRNA levels, using data available from the Kocak dataset. The mRNA levels of *PRMT1* significantly correlated with the *MYCN* transcripts (Supplementary Figure S1C), consistent with the notion that MYCN regulates *PRMT1* expression. As the mRNA transcripts are not necessarily correlated with protein expression levels, we sought to further examine PRMT1 and MYCN protein expression by immunohistochemistry in neuroblastoma tissue microarrays containing 98 patient samples [7]. We found that PRMT1 protein levels were significantly higher in neuroblastomas than in ganglioneuroma (GN) and ganglioneuroblastoma (GNB), two types of low-risk peripheral neuroblastic tumors consisting of differentiated, stroma-rich ganglion cells (*P* < 0.01; Figure 1A–1B). In this series of 66 neuroblastomas, 37 showed a high level of PRMT1 protein expression and 29 showed negative or low PRMT1 expression (Figure 1B). Among neuroblastomas with high PRMT1 levels, 62% (*n* = 23) showed high MYCN protein expression (Figure 1C–1D). This relationship was also seen in a panel of neuroblastoma cell lines (Figure 1E). The strong correlations between MYCN and PRMT1 at both mRNA and protein levels imply that these two proteins may form a functional MYCN-PRMT1 axis in neuroblastoma.

### PRMT1 physically interacts with MYCN

**Figure 2:**
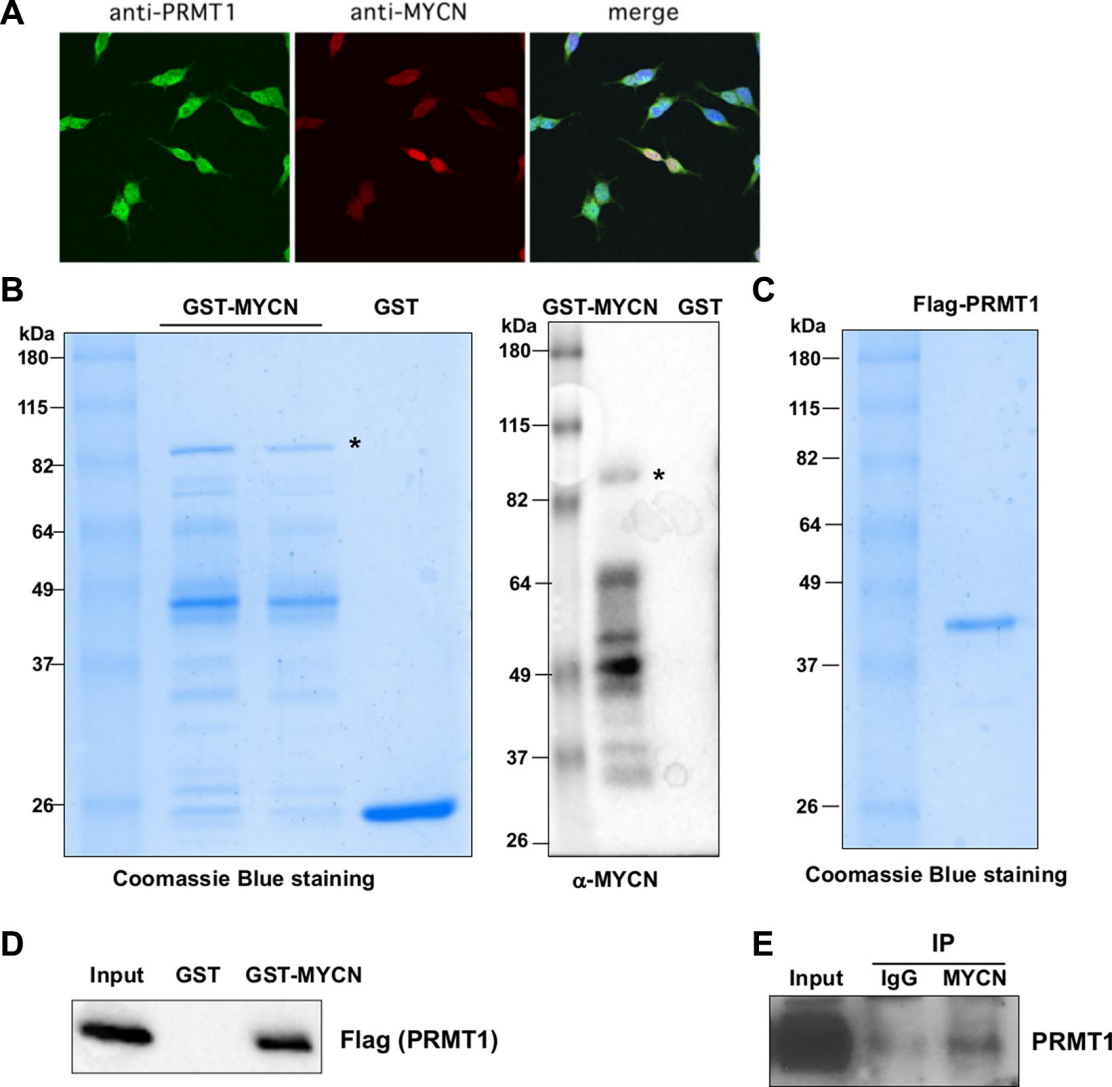
PRMT1 interacts with MYCN (**A**) Colocalization of MYCN and PRMT1 as shown by confocal images of immunostaining of PRMT1 and MYCN in LAN-5 cells. (**B**) Coomassie blue staining of bacterially expressed GST-MYCN (left). A full western blot of GST and GST-MYCN as detected by anti-MYCN antibody (right). (**C**) Coomassie blue staining of recombinant PRMT1 protein expressed from Sf9 insect cells. (**D**) MYCN directly binds to PRMT1 *in vitro*. GST pull-down assays were performed using Flag-PRMT1 with GST or GST-MYCN followed by immunoblotting with anti-Flag antibody. (**E**) Endogenous MYCN physically interacts with PRMT1. Nuclear extracts from LAN-5 cells were subjected to CoIP analysis with α-MYCN antibody, followed by immunoblotting with α-PRMT1 antibody.

To gain insight into the possible mechanistic link between MYCN and PRMT1, we sought to determine whether MYCN and PRMT1 can be physically associated to form a protein complex. To this end, we first conducted immunostaining assays with specific MYCN and PRMT1 antibodies using *MYCN*-amplified LAN-5 cells. As shown in Figure 2A, the double immunostaining of MYCN and PRMT1 favors a significant co-localization pattern in LAN-5 cells, suggesting that endogenous MYCN and PRMT1 proteins may form a protein complex and this complex is localized to the nucleus. To directly test the potential interaction between MYCN and PRMT1, we first tested direct binding of MYCN and PRMT1 *in vitro*. Bacterially expressed GST-MYCN fusion protein was purified and then verified by western blot analysis with specific anti-MYCN antibody (Figure 2B). GST pull-down assays showed that GST-MYCN, but not GST control, specifically bound to Flag-tagged recombinant PRMT1 protein purified from baculovirus-infected insect cells (Figure 2C–2D). To further examine whether there is a physical association between endogenous PRMT1 and MYCN in neuroblastoma cells, their interactions were assessed following immunoprecipitation in LAN-5 cells. Nuclear extracts were prepared and subjected to immunoprecipitation with an anti-MYCN monoclonal antibody. Figure 2E demonstrates that endogenous MYCN forms a complex with PRMT1 in neuroblastoma cells carrying *MYCN* amplification. Together, the observations of colocalization and physical interaction of MYCN and PRMT1 suggest a potential functional role of this novel protein complex in neuroblastoma.

### PRMT1 is a novel regulator of MYCN expression

**Figure 3:**
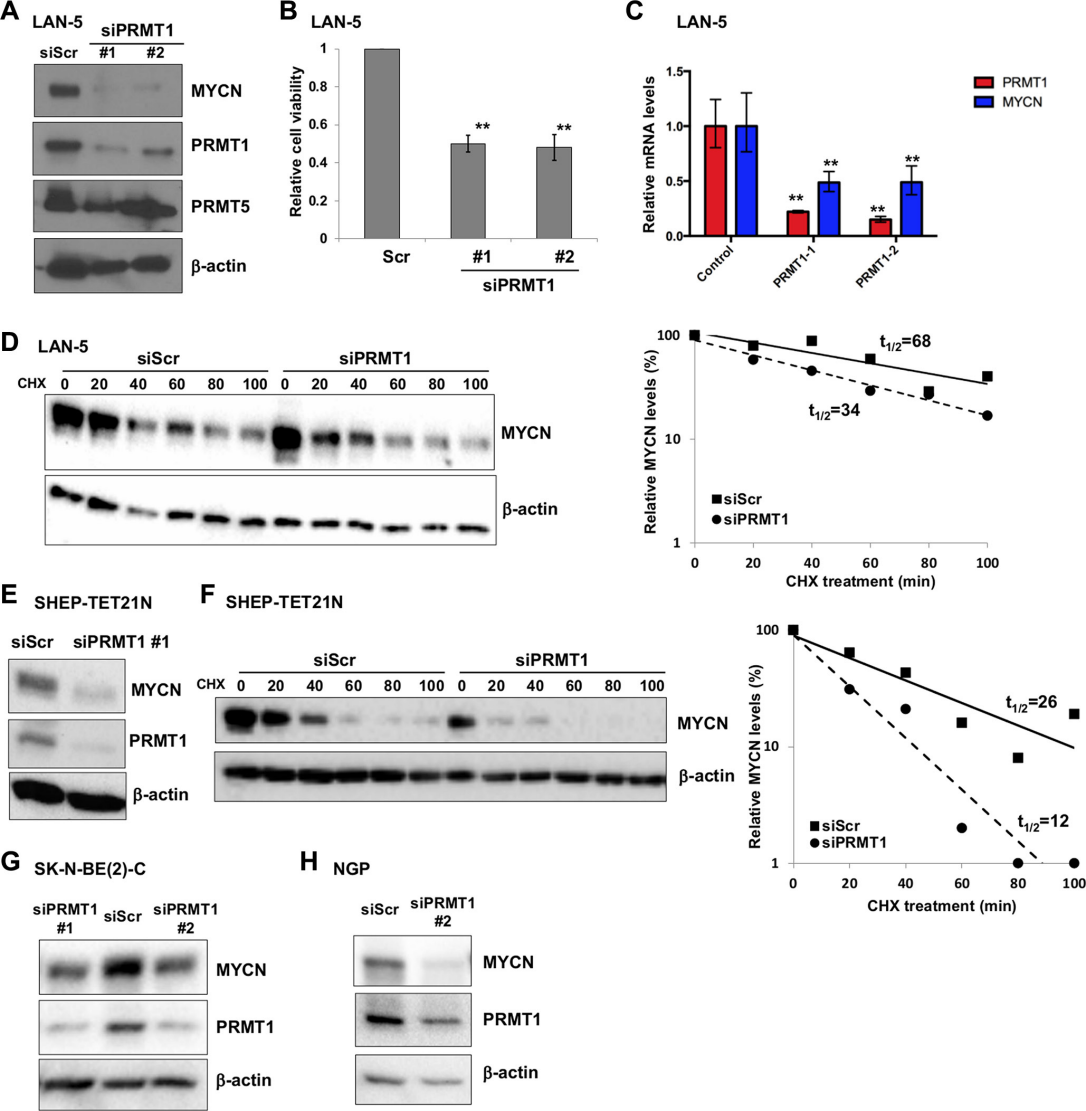
PRMT1 regulates MYCN expression (**A**) Western blot analysis of PRMT1 knockdown in LAN-5 cells 6 days following transfection with two independent PRMT1 siRNAs with indicated antibodies. β-actin was used as a loading control. (**B**) PRMT1 knockdown reduces cell viability. Cell viability was measured using the Trypan blue viability test and results are expressed as percentage of viable cells. Mean ± SEM (*n* = 3, Student's *t* test, **P < 0.01). (**C**) RT-qPCR analysis of *PRMT1* and *MYCN* mRNA levels in siPRMT1-transfectd LAN-5 cells. Mean ± SEM (*n* = 3, Student's *t* test, **P < 0.01). (**D**) Immunoblots with anti-MYCN and β-actin (loading control) after CHX treatment following transient transfection of scrambled siRNA (siScr) or siPRMT1#1 in LAN-5 cells. Plots of densitometric quantification of MYCN protein stability were shown. (**E**) Western blot analysis of PRMT1 knockdown in SHEP-TET21N cells (Tet-off, MYCN on) 6 days following transfection with siPRMT1 #1 with indicated antibodies. β-actin was used as a loading control. (**F**) Immunoblots with anti-MYCN and β-actin after CHX treatment following transient transfection of scrambled siRNA (siScr) or siPRMT1#1 in SHEP-TET21N cells (Tet-off, MYCN on). Plots of densitometric quantification of MYCN protein stability were shown. Western blot analysis of PRMT1 knockdown in SK-N-BE(2)-C (**G**) and NGP cells (**H**) 6 days following transfection with PRMT1 siRNAs.

The correlated expression and physical association between MYCN and PRMT1 motivated us to explore the possible functions of the MYCN-PRMT1 axis. To directly examine the role of PRMT1, we evaluated the effect of short-interfering RNA- (siRNA-) mediated PRMT1 depletion on MYCN expression. PRMT1 knockdown led to a dramatic decrease of MYCN protein levels with two independent PRMT1 siRNAs (Figure 3A), accompanied by a significant reduction of cell viability (Figure 3B). Since PRMT5 was recently shown to regulate MYCN expression [8], we investigated whether PRMT1 indirectly influences MYCN expression through PRMT5. As shown in Figure 3A, PRMT1 knockdown did not result in appreciable changes of PRMT5 expression. Importantly, *MYCN* mRNA levels were also markedly decreased following PRMT1 depletion (Figure 3C), suggesting that PRMT1 may regulate MYCN transcription. Additional experiments, such as chromatin immunoprecipitation, gel shift, reporter assay, and mutational analysis of MYCN promoter, are needed to define the binding mode of PRMT1 at MYCN promoter and the mechanism of transcriptional regulation. Given the fact that we have previously identified PRMT1 as a MYCN target gene [3], our observations so far suggest that PRMT1 also regulates MYCN expression through a forward feedback loop.

To evaluate at what levels PRMT1 influences MYCN expression, we measured the MYCN half-life by treating LAN-5 cells with cycloheximide (CHX) to block new protein synthesis, and cells were harvested at different time points afterward. As shown in Figure 3D, MYCN turned over with a half-life of 68 min after CHX treatment in LAN-5 cells transfected with scrambled control siRNA, whereas PRMT1 knockdown markedly reduced MYCN half-life to approximately 34 min. We also used the SHEP-TET21N model system in which ectopic MYCN expression is induced following removal of tetracycline [9]. Depletion of PRMT1 led to a marked reduction of ectopically expressed MYCN (Figure 3E). Importantly, we measured the MYCN half-life by treating cells with CHX. MYCN turned over with a half-life of 26 min in SHEP-TET21N cells transfected with scrambled control siRNA, consistent with a previous report [10]. PRMT1 knockdown markedly reduced MYCN half-life to approximately 12 min (Figure 3F). Notably, we observed downregulation of MYCN in two additional, independent *MYCN*-amplified neuroblastoma cell lines SK-N-BE(2)-C and NGP when PRMT1 was depleted (Figure 3G–3H). Together, our data suggest that PRMT1 may regulate MYCN protein expression by two discrete mechanisms, one possibly at the transcriptional level (Figure 3C) and the other at the post-translational level (Figure 3D–3F). This is reminiscent of the regulatory relationship between PRMT1 and c-MYC: PRMT1 activates the transcriptional activity of c-MYC promoter through modulation of chromatin structure [11], whereas c-MYC directly activates PRMT1 promoter by binding to putative c-MYC binding sites [12]. The exact mechanisms behind this positive regulatory expression loop involving PRMT1 and MYC family proteins (c-MYC and MYCN) await future investigation.

### PRMT1 methylates MYCN

**Figure 4:**
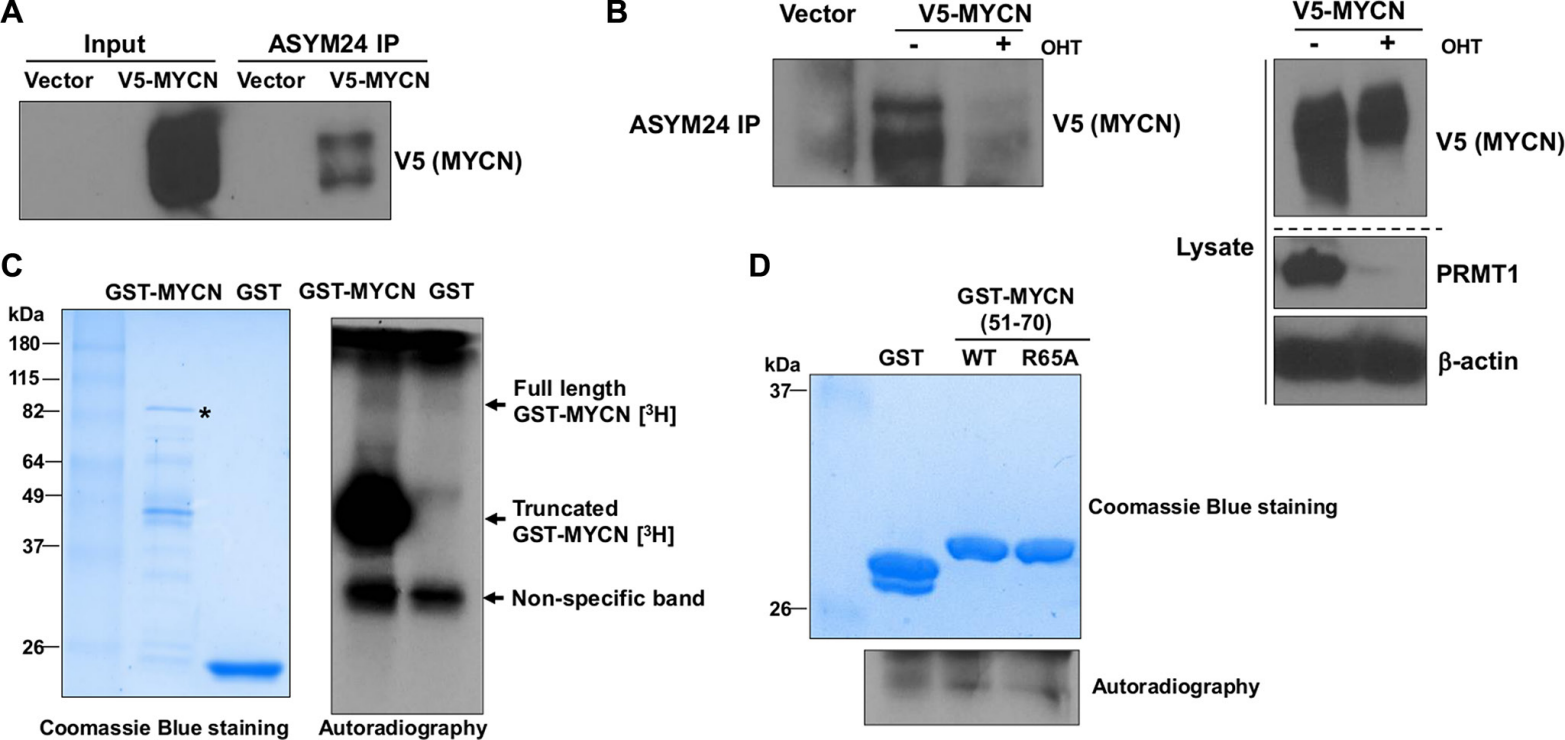
PRMT1 methylates MYCN (**A**) *Prmt1^fl/−^ER-Cre* MEF cells were transfected with pcDNA3.1 V5-MYCN. Two days after transfection, the whole cell lysates were subjected to immunoprecipitation with ASYM24 antibody, followed by western blot with anti-V5 antibody. (**B**) (left) *Prmt1^fl/−^ER-Cre* MEF cells were induced with OHT for 4 days, followed by transfection with pcDNA3.1 V5-MYCN for 2 days, and analyzed for IP with ASYM24 antibody. (right) Western blot analysis of whole cell lysates with anti-V5 and anti-PRMT1 (in a separate repeat blot) antibodies. β-actin was used as a loading control. (**C**) *In vitro* methylation assays with recombinant PRMT1 together with GST or GST-MYCN full-length proteins in the presence of [^3^H] SAM. A non-specific band close to 37 kDa is indicated, most likely derived from co-purified proteins with recombinant PRMT1 from insect cells. (**D**) *In vitro* methylation assays with recombinant PRMT1 together with GST or GST-MYCN (51–70) fusion proteins (WT or R65A) in the presence of [^3^H] SAM.

MYCN is known to undergo a number of post-translational modifications. However, post-translational arginine methylation of MYCN has not been previously appreciated. A recent study identified several potential sites of arginine methylation on MYCN protein by using liquid chromatography tandem mass spectrometry (LC-MS/MS) analysis [8]. As it is not possible to discern asymmetrical and symmetrical dimethylation from the previous analysis [8], we wished to assess whether MYCN may be asymmetrically dimethylated by PRMT1. It has been shown that the epitope for ASYM24, an antibody that specifically recognizes asymmetrical dimethylated arginine-containing cellular proteins, diminishes significantly in PRMT1 knockout cells, indicating that PRMT1 is the major enzyme that contributes to the ASYM24 epitope [13]. As shown in Figure 4A, transiently transfected V5-tagged MYCN was detected in the ASYM24 immunoprecipitate from floxed MEF cell line (*Prmt1^fl/−^ER-Cre*) [14], indicating that MYCN is asymmetrically dimethylated in cultured cells. To determine whether PRMT1 is the major enzyme catalyzing MYCN asymmetrical demethylation, we induced *Prmt1* knockout in *Prmt1^fl/−^ER-Cre* MEF cells. As shown in Figure 4B, treatment with 4-hydroxytamoxifen (OHT) dramatically reduced the methylation levels of V5-tagged MYCN protein. Notably, OHT treatment induced a highly efficient *Prmt1* loss, accompanied by a decrease of the expression levels of V5-MYCN (Figure 4B). These data provide strong evidence that MYCN represents a novel substrate of PRMT1, implying that PRMT1 is a key regulator of MYCN protein by arginine methylation.

PRMT1 is known to recognize and methylate arginine residues within glycine-arginine-rich (GAR) motifs (15). We next searched for putative PRMT1-methylation GAR motifs in the entire MYCN sequence and found several GAR domains (data not shown). The high degree of sequence conservation in one of these domains prompted us to conduct *in vitro* methylation assays to test whether R65 is a major arginine site for PRMT1-mediated methylation. Recombinant GST or GST-MYCN full length proteins were incubated with purified PRMT1 and^3^H-S-AdoMet (^3^H-SAM), a methyl group donor. GST-MYCN fusion protein, but not GST, was methylated by PRMT1 (Figure 4C). Furthermore, we expressed MYCN (51-70) fragments as GST fusion proteins and conducted *in vitro* methylation. As shown in Figure 4D, mutating arginine 65 to alanine markedly reduced the methylation signal. These observations confirmed that R65 is one of the primary sites for *in vitro* methylation of MYCN by PRMT1.

### Mutating methylated R65 impairs MYCN stability

**Figure 5:**
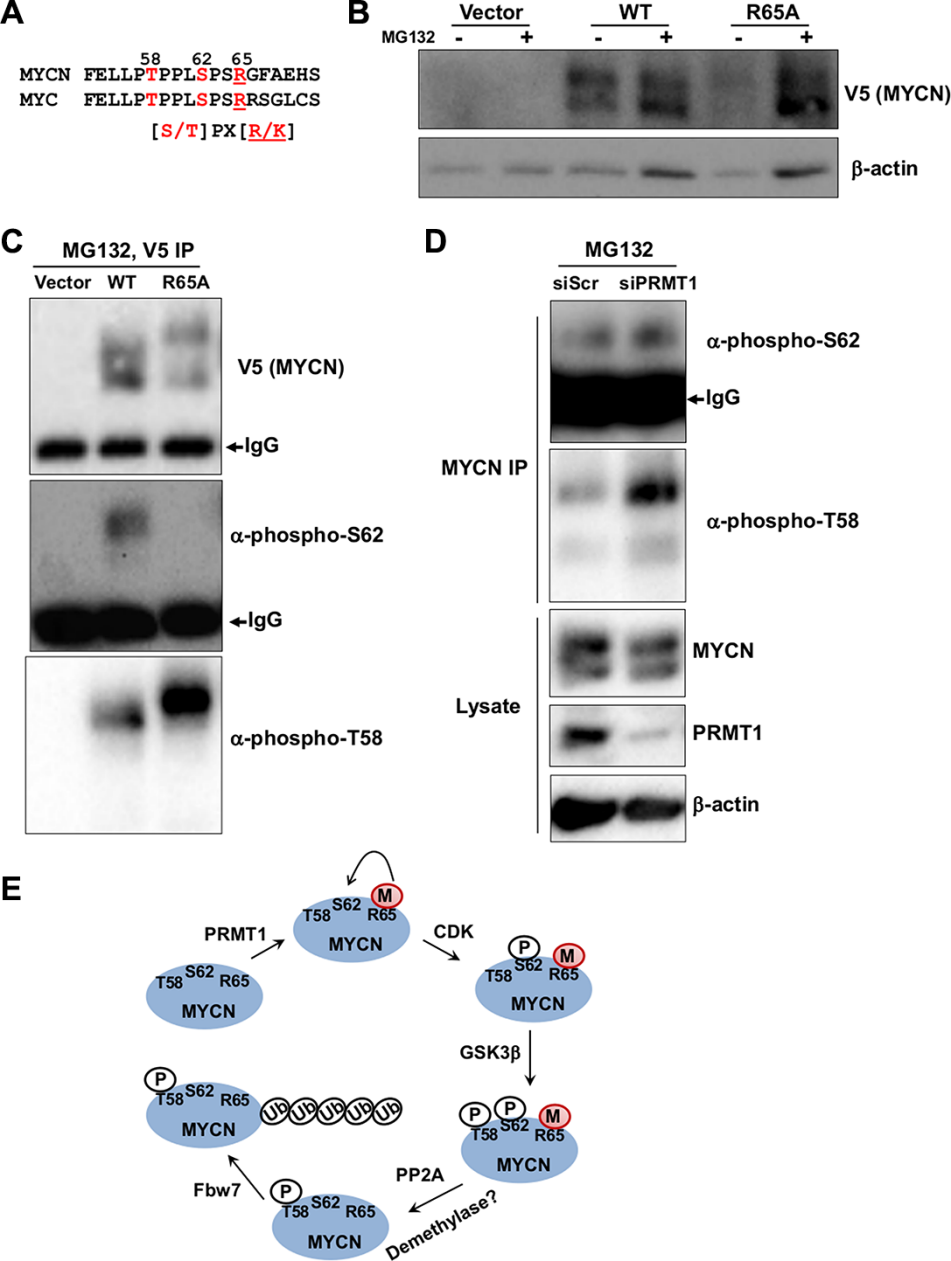
Cross-talk between R65 methylation and pT58 and pS62 (**A**) Alignment of MYCN (53-71) and corresponding MYC protein sequences. The consensus substrate sequence of CDK is also shown. (**B**) Western blot analysis of stably transfected HEK293 cells with V5-tagged MYCN wildtype or R65A in the presence of absence of MG132 treatment (6 hr). β-actin was used as a loading control. (**C**) V5-tagged MYCN was immunoprecipitated with anti-V5 antibody from (B) after MG132 treatment, followed by western blot with indicated antibodies. The IgG heavy-chain was also indicated. (**D**) MYCN was immunoprecipitated with anti-MYCN antibody from SHEP-TET21N cells (Tet-off, MYCN on) 3 days post-transfection with siPRMT1#1 and 6 hr of MG132 treatment, followed by western blot with indicated antibodies. Western blot analysis of whole cell lysates was also shown. β-actin was used as a loading control. (**E**) The life cycle of MYCN from synthesis to degradation is shown. PRMT1-mediated R65 methylation may prime MYCN for phosphorylation at S62 via CDK, stabilizing MYCN and further priming it for phosphorylation at T58 through GSK3β. Dephosphorylation of pS62 via protein phosphatase 2A (PP2A) sensitizes MYCN phosphorylated at T58 to bind to E3 ligase, such as F-box and WD repeat domain-containing 7 (Fbw7), for subsequent ubiquitination and degradation. Demethylation of R65 may occur through unknown demethylases.

It has been long known that a complex signaling pathway affects MYC/MYCN stability through sequential phosphorylation of two highly conserved sites, S62 and T58. Phosphorylation at S62 (pS62) can stabilize MYC, whereas subsequent phosphorylation at T58 (pT58) promotes MYC proteolysis [16]. While ERK or CDK kinases have been identified as the kinases for phosphorylation at S62, the relative importance and underlying mechanism of local sequence context in kinases activity remain unknown [17]. The observation that the C-terminal arginine/lysine residues in the consensus motif of CDK substrates, including MYC/MYCN, are essential for their phosphorylation at S/T [17; Figure 5A] has prompted us to hypothesize that arginine methylation at R65 may cross-regulate CDK-mediated phosphorylation at S62. To this end, we created HEK293 cells stably transfected with V5-tagged MYCN expression constructs. As shown in Figure 5B, MYCN R65A mutant has lower level of expression, as compared to wildtype MYCN. Consistent with enhanced proteasome-mediated turnover in R65A mutant, the proteasome inhibitor MG132 stabilized R65A significantly to a level comparable to wildtype MYCN. Next, we investigated whether alteration of the MYC degradation pathway involving T58 and S62 phosphorylation could account for the decreased MYCN stability in R65A mutant. We examined phosphorylation at T58 and S62 using phospho-specific antibodies in the immunoprecipitate of V5-MYCN after MG132 treatment. We found less pS62 and more pT58 in the R65A MYCN mutant compared to wildtype MYCN (Figure 5C), suggesting increased degradation. We consistently noticed that the mobility of R65A MYCN mutant did not line up with that of MYCN WT (Figure 5C V5-MYCN and phospho-T58 panels). It is possible that R65A mutation may lead to increased levels of pT58 and subsequent alterations in protein charge and/or structure that cause a mobility shift. To investigate the role of PRMT1 in regulation of MYCN stability, we then performed similar analysis in SHEP-TET21N cells upon siRNA-mediated PRMT1 knockdown. Consistently, we saw increased p-T58 MYCN upon MG132 treatment in PRMT1-depleted cells. Taken together, these data demonstrate that PRMT1 can increase MYCN expression and stability, at least in part, through altering T58 and S62 phosphorylation, providing a biological relevance of increased PRMT1 expression in MYCN-expressing neuroblastomas. We propose that methylation of MYCN R65 promotes MYCN stabilization through CDK-mediated phosphorylation of S62 (Figure 5E). Although the detailed molecular mechanisms of this cross-talk await further clarification, our data suggest a very prominent role of R65, likely through PRMT1-mediated arginine methylation, in orchestrating the key phosphorylation events that alter MYCN protein stability (Figure 5C–5E).

MYCN plays a causative role in neuroblastoma and direct targeting of MYCN has been an elusive goal of many cancer drug development efforts. The current model of MYCN function in neuroblastoma implies that MYCN regulates a complex network to drive neuroblastoma tumorigenesis, underscoring the need to uncover key critical molecular targets and pathways for therapeutic intervention based on MYCN transcriptomic signature. Here we demonstrate that PRMT1, a MYCN target, is a critical regulator of MYCN expression. PRMT1 and MYCN expression act in a positive feedback loop in neuroblastoma cells. These results highlight the MYCN-PRMT1-MYCN oncogenic axis in neuroblastoma and suggest that targeting PRMT1 represents a promising treatment approach for MYCN-driven neuroblastoma.

## MATERIALS AND METHODS

### Neuroblastoma TMAs and patient samples

Neuroblastoma TMAs were obtained from COG Biospecimen Repository (Nationwide Children's Hospital, Columbus, OH). Normal tonsil tissue as well as ganglioneuroma/ganglioneuroblastoma tumor tissue samples were included in the TMAs as control tissue. The neuroblastoma NB122 set from patients with neuroblastoma of all stages was described previously [3].

### Analyses of patient data

Patient data used in this study were described previously [18]. Gene expression datasets were obtained from the R2 microarray analysis and visualization platform (http://hgserver1.amc.nl/cgi-bin/r2/main.cgi?&species=hs). Kaplan–Meier analysis was conducted online, and the resulting survival curves and *P* values (log-rank test) were downloaded. All cutoff values for separating high and low expression groups were determined by the online algorithm.

### Cell lines, plasmids, transfection and treatment

Neuroblastoma cell lines SH-EP1, SH-SY5Y, KELLY, SK-N-BE(2)-C and IMR32, SK-N-AS were purchased from Sigma and ATCC, respectively. LAN-6, SMS-LHN, CHLA-90, LAN-5 and SMS-KCNR cell lines were obtained from COG (http://www.cogcell.org). NGP was purchased from DSMZ (Braunschweig, Germany). The MYCN-inducible SHEP-TET21N cells were generously provided by Dr. M. Schwab (DKFZ, Heidelberg, Germany, 9). MYCN expression was induced by removal of tetracycline (1 μg/ml) from the growth medium. All of these cell lines were cultured according to standard protocols. The Prmt1^*fl/−*^ER*-Cre* MEF cells were generously provided by Dr. Stephane Richard (McGill University, Montreal, Canada) and maintained as previously described [14]. These MEFs were treated with 3 μM OHT for 5 days to generate *Prmt1* KO MEFs. Cycloheximide and MG132 were purchased from Sigma and the working concentrations are 50 μg/ml and 10 μg/ml (6 hr), respectively. Cell viability was determined using Trypan blue staining.

The human MYCN cDNA expression construct was subcloned from pTH-MYCN, a gift from William A. Weiss (Addgene plasmid # 35416) [19], into pcDNA 3.1 vector to make pcDNA3.1-V5-MYCN, and into pGEX-5X-1 to make pGEX-5X-V5-MYCN. A QuikChange site-directed mutagenesis kit (Stratagene) was used to generate point mutations of MYCN. The MYCN (51-70) fragments were PCR amplified and subcloned into pGEX-5X-1.

For short-interfering RNA (siRNA) treatments, exponentially growing cells were transfected using Nucleofector kits (Amaxa) for LAN-5 cells and RNAiMax (Invitrogen) for other cells at settings recommended by the manufacturer. Cells were lysed for protein or for RNA analysis at indicated time points post-transfection. Target sequences of siRNAs are: GACATGACATCCAAAGATTAC and CACCATCGACCTGGACTTCAA. Transfection of V5-MYCN in MEF cells was carried out using jetPRIME™ (Poly-plus Transfection, France) per manufacturer's protocol. Stable HEK293 cells were selected in the presence of 500 μg/ml G418 for 2 weeks post-transfection with Lipofectamine 2000 (Invitrogen).

### Immunohistochemistry

Immunohistochemical studies were performed essentially as previously described [7]. Briefly, prior to immunostaining tissue underwent deparaffinization in xylene, followed by antigen retrieval, and blocking of endogenous peroxidase. The immunohistochemical staining was done using a standard labeled streptavidin-biotin method (VECTASTAINVR ABC Kit, # PK-4001, Vector Laboratories, CA, USA) followed by 3,3′-diaminobenzidine enzymatic development. The following antibodies were used: anti-PRMT1 antibody (Millipore, #07-404, 1:100 dilution) and anti-MYCN antibody (Santa Cruz, #sc-53993, 1:100 dilution). Normal rabbit or mouse IgG serves as the negative control. Slides were imaged with an Olympus VS120 slide scanner system. Immunoreactivity for PRMT1 and MYCN was independently scored by two investigators (JH and LL), both blinded as to the identity of each specimen, based on the previously described scoring method [7]. The statistical analysis was done by a two-tailed Fisher's exact test with a 95% confidence interval; *P* values < 0.05 were considered significant.

### Immunoblotting, co-immunoprecipitation, protein purification, GST pull-down and *in vitro* methylation assays

Isolation of whole cell lysates and nuclear extracts, immunoblotting, co-immunoprecipitation, purification of baculovirus-infected insect cells and GST fusion proteins, GST pull-down and *in vitro* methylation assays were performed as previously described [5]. Antibodies used in this study include anti-PRMT1 (Millipore #07-404), anti-MYCN (Santa Cruz #sc-53993), anti-PRMT5 (Millipore #07-405), anti-β-actin (Sigma #A5316), anti-V5 (Invitrogen #R960-25), anti-phospho-T58 MYC (Applied Biological Material #Y011034), anti-phospho-S62 MYC (BioAcademia #E71-161) and anti-ASYM24 (Millipore #07-414).

### Gene expression analysis

Total RNA was extracted using the RNeasy Plus mini kit (QIAGEN) and quantitative real-time reverse transcription-PCR (qRT-PCR) was performed as previously described [5]. The primers and probes used in this study are: MYCN (Applied Biosystems # Hs00232074_m1), PRMT1 (Applied Biosystems # Hs01587651_g1) and HPRT1 (Applied Biosystems # 4369016).

## SUPPLEMENTARY MATERIALS FIGURE



## References

[1] Louis CU, Shohet JM (2015). Neuroblastoma: molecular pathogenesis and therapy. Annu Rev Med.

[2] Barone G, Anderson J, Pearson AD, Petrie K, Chesler L (2013). New strategies in neuroblastoma: Therapeutic targeting of MYCN and ALK. Clin Cancer Res.

[3] Valentijn LJ, Koster J, Haneveld F, Aissa RA, van Sluis P, Broekmans ME, Molenaar JJ, van Nes J, Versteeg R (2012). Functional MYCN signature predicts outcome of neuroblastoma irrespective of MYCN amplification. Proc Natl Acad Sci U S A.

[4] Yang Y, Bedford MT (2013). Protein arginine methyltransferases and cancer. Nat Rev Cancer.

[5] Li X, Hu X, Patel B, Zhou Z, Liang S, Ybarra R, Qiu Y, Felsenfeld G, Bungert J, Huang S (2010). H4R3 methylation facilitates beta-globin transcription by regulating histone acetyltransferase binding and H3 acetylation. Blood.

[6] Shia WJ, Okumura AJ, Yan M, Sarkeshik A, Lo MC, Matsuura S, Komeno Y, Zhao X, Nimer SD, Yates JR, Zhang DE (2012). PRMT1 interacts with AML1-ETO to promote its transcriptional activation and progenitor cell proliferative potential. Blood.

[7] Hansen JN, Lotta LT, Eberhardt A, Schor NF, Li X (2016). EYA1 expression and subcellular localization in neuroblastoma and its association with prognostic markers. J Cancer Res Therapy.

[8] Park JH, Szemes M, Vieira GC, Melegh Z, Malik S, Heesom KJ, Von Wallwitz-Freitas L, Greenhough A, Brown KW, Zheng YG, Catchpoole D, Deery MJ, Malik K (2015). Protein arginine methyltransferase 5 is a key regulator of the MYCN oncoprotein in neuroblastoma cells. Mol Oncol.

[9] Lutz W, Stöhr M, Schürmann J, Wenzel A, Löhr A, Schwab M (1996). Conditional expression of N-myc in human neuroblastoma cells increases expression of alpha-prothymosin and ornithine decarboxylase and accelerates progression into S-phase early after mitogenic stimulation of quiescent cells. Oncogene.

[10] Slamon DJ, Boone TC, Seeger RC, Keith DE, Chazin V, Lee HC, Souza LM (1986). Identification and characterization of the protein encoded by the human N-myc oncogene. Science.

[11] Rezai-Zadeh N, Zhang X, Namour F, Fejer G, Wen YD, Yao YL, Gyory I, Wright K, Seto E (2003). Targeted recruitment of a histone H4-specific methyltransferase by the transcription factor YY1. Genes Dev.

[12] Fujimoto K, Matsuura K, Hu-Wang E, Lu R, Shi YB (2012). Thyroid hormone activates protein arginine methyltransferase 1 expression by directly inducing c-Myc transcription during Xenopus intestinal stem cell development. J Biol Chem.

[13] Boisvert FM, Côté J, Boulanger MC, Richard S (2003). A proteomic analysis of arginine-methylated protein complexes. Mol Cell Proteomics.

[14] Yu Z, Chen T, Hébert J, Li E, Richard S (2009). A mouse PRMT1 null allele defines an essential role for arginine methylation in genome maintenance and cell proliferation. Mol Cell Biol.

[15] Bicker KL, Obianyo O, Rust HL, Thompson PR (2011). A combinatorial approach to characterize the substrate specificity of protein arginine methyltransferase 1. Mol Biosyst.

[16] Farrell AS, Sears RC (2014). MYC degradation. Cold Spring Harb Perspect Med.

[17] Alexander J, Lim D, Joughin BA, Hegemann B, Hutchins JR, Ehrenberger T, Ivins F, Sessa F, Hudecz O, Nigg EA, Fry AM, Musacchio A, Stukenberg PT (2011). Spatial exclusivity combined with positive and negative selection of phosphorylation motifs is the basis for context-dependent mitotic signaling. Sci Signal.

[18] Kocak H, Ackermann S, Hero B, Kahlert Y, Oberthuer A, Juraeva D, Roels F, Theissen J, Westermann F, Deubzer H, Ehemann V, Brors B, Odenthal M (2013). Hox-C9 activates the intrinsic pathway of apoptosis and is associated with spontaneous regression in neuroblastoma. Cell Death Dis.

[19] Weiss WA, Aldape K, Mohapatra G, Feuerstein BG, Bishop JM (1997). Targeted expression of MYCN causes neuroblastoma in transgenic mice. EMBO J.

