# Supplementary material for: Protein arginine methyltransferase 1 is a novel regulator of MYCN in neuroblastoma
Source: Oncotarget. 2016 Aug 23;7(39):63629–39. doi: 10.18632/oncotarget.11556 (PMC5325390; doi:10.18632/oncotarget.11556)
Supplement: Supplementary file 1 [file oncotarget-07-63629-s001.pdf]

Protein arginine methyltransferase 1 is a novel regulator of MYCN in neuroblastoma

Supplementary Materials

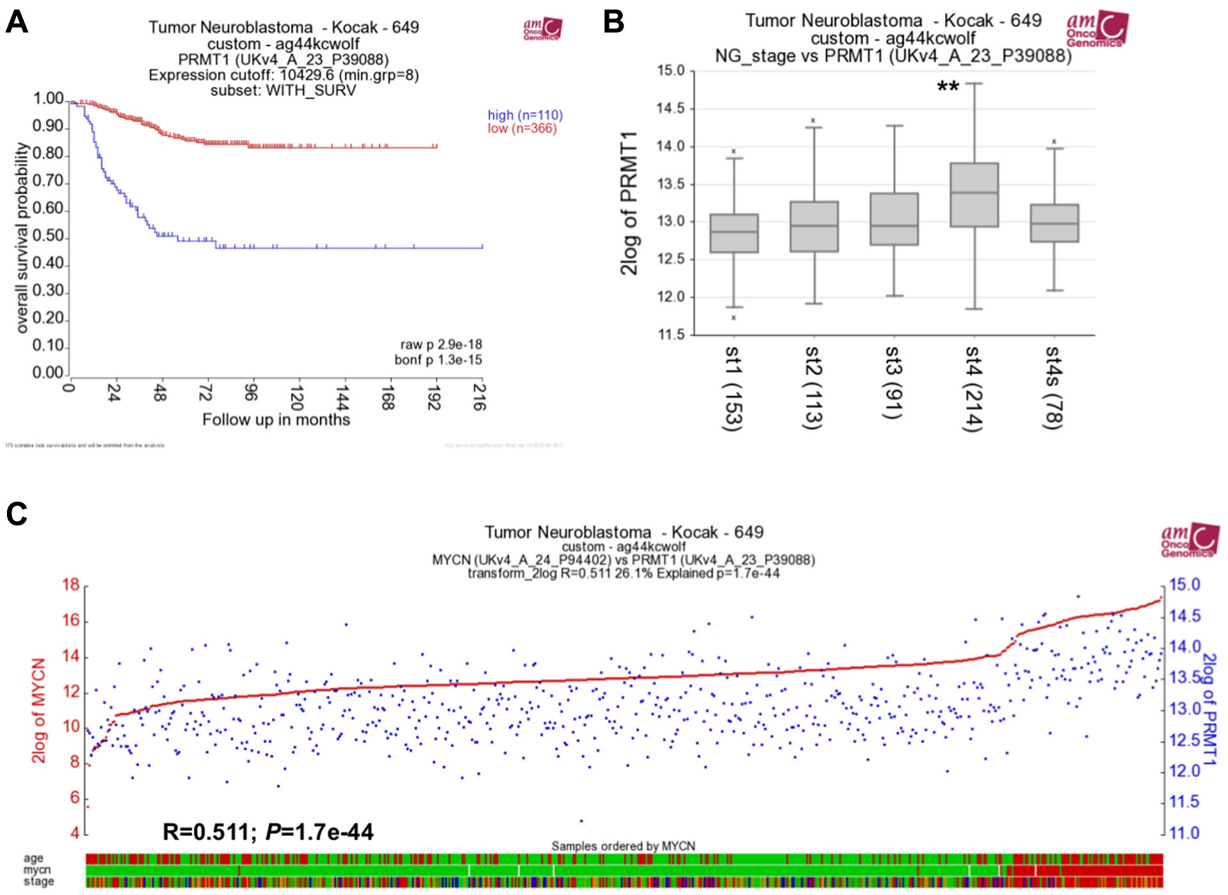

Supplementary Figure S1: *PRMT1* mRNA expression as a significant independent prognostic marker in primary neuroblastomas. (**A**) High *PRMT1* expression is prognostic for poor survival and aggressive behavior in neuroblastoma patients. Kaplan-Meier analysis of overall survival for the Kocak dataset. The graph was downloaded from R2 microarray analysis and visualization platform (http://r2.amc.nl). Patients with higher *PRMT1* expression are highlighted in blue, whereas patients with lower *PRMT1* expression are highlighted in red. The log-rank test *P* values are indicated. (**B**) Box plot of *PRMT1* expression levels in stage (ST) 1-4S tumors. One-way analysis of variance (ANOVA), $^{**}P < 0.01$. (**C**) YY plot showing the correlation of *MYCN* (red) and *PRMT1* (blue) mRNA levels in neuroblastoma tumor series Kocak dataset. Below the graph tracks for age, *MYCN* amplification, and International Neuroblastoma Staging System stages are shown.
